# Infrared Gas Detection Method Based on Non-Solid Characteristics and Spatiotemporal Information

**DOI:** 10.3390/s26113284

**Published:** 2026-05-22

**Authors:** Xin Zhang, Shiwei Xu

**Affiliations:** 1 Marine Design and Research Institute of China, South Xizang Road, Shanghai 200011, China; 2 Key Laboratory of Photoelectronic Imaging Technology and System, Ministry of Education, Beijing Institute of Technology, Beijing 100081, China; shiwei.xu@raysense.com

**Keywords:** image processing, gas leak detection, infrared imaging, non-solid characteristics, spatiotemporal information

## Abstract

Infrared imaging technology has been widely adopted for industrial gas leak detection due to its capability for large field-of-view, long-range, and dynamic monitoring. However, in practical applications, natural object interference within the scene, together with the blurred contours and low contrast of infrared images, severely degrades the performance of gas detection and leakage region segmentation. To address these challenges, this paper proposes a gas leak detection method that integrates gas characteristics with spatiotemporal information. Specifically, the non-solid characteristics of gas are incorporated to constrain the foreground extraction process of the Gaussian Mixture Model (GMM), thereby suppressing interfering moving objects. Furthermore, by exploiting the spatiotemporal information in infrared image sequences, a multi-scale cross-attention fusion model is designed to fuse multi-scale and global feature representations, improving the accuracy of foreground detection. Finally, density-based clustering is employed to achieve complete segmentation of gas regions with irregular shapes. Experimental results demonstrate that the proposed method effectively suppresses interference from solid objects, accurately detects gas leakage, and successfully segments the diffusion regions. Compared with existing approaches, the proposed method shows significant advantages and provides a valuable reference for research on infrared imaging-based gas leak detection.

## 1. Introduction

Industrial gases play a vital role in key sectors of social and economic development, including steel manufacturing, commercial operations, transportation, and the energy and chemical industries, and have become an important component of modern energy systems [1]. However, during the storage, utilization, and transportation of industrial gases, leakage incidents occur frequently. Such incidents not only cause severe environmental pollution but may also lead to major safety hazards such as fires, explosions, and toxic exposure [2]. Gas leak detection technologies are capable of establishing a comprehensive safety assurance system throughout the entire lifecycle of leakage events. These technologies enable timely early warnings at the initial stage of leakage, facilitate orderly evacuation guidance during the intermediate stage, and help reduce secondary disaster losses. In addition, they provide complete data records for post-incident analysis and investigation. Furthermore, achieving the goals of carbon peaking and carbon neutrality [3] has become a strategic requirement for sustainable and green development in China. Therefore, developing techniques that can rapidly and accurately detect gas leaks and effectively identify leakage regions and diffusion ranges is of great significance for ensuring industrial safety and reducing accident risks.

Traditional gas detection technologies can generally be categorized into two types. The first type relies on the large-scale deployment of single-point sensors within the monitoring area [4,5]. Such methods exhibit high sensitivity, reaching parts-per-million (ppm) levels. However, they suffer from high maintenance costs and are susceptible to signal drift caused by wind direction variations. The second type consists of sampling-based analytical detection techniques, such as gas chromatography [6,7]. Although these methods can provide information on gas composition and concentration within sampled regions, they are unable to monitor the dynamic evolution of gas leaks in real time and typically suffer from significant response delays. Consequently, both approaches exhibit limitations in rapidly locating leakage sources and predicting leakage dynamics. Infrared imaging technology, owing to its advantages of non-contact detection, long-range monitoring, and intuitive visualization [8,9], has gradually become an important tool for industrial gas leak detection. However, in real-world scenarios, uncertainties in environmental factors such as temperature, humidity, and wind speed, together with the non-solid nature and uncertain characteristics of gas targets [10], as well as noise interference, often lead to blurred boundaries and low contrast of gas targets in infrared images. As a result, developing effective interference suppression and target detection algorithms for infrared imaging in complex environments, particularly for low-contrast gas detection and region segmentation, has become an important research topic.

Existing infrared gas detection methods can generally be divided into two categories: motion-based target detection methods and deep learning-based detection methods. Deep learning approaches have the advantage of learning complex features of targets directly from raw images through large-scale datasets, enabling real-time monitoring of gas leakage. Wang et al. [11] collected and annotated the first large-scale methane leak video dataset, GasVid, and evaluated the detection performance of convolutional neural networks (CNNs) with different levels of complexity. Their results achieved nearly 100% accuracy in leak/non-leak classification. However, this work focused only on determining the occurrence of gas leakage without addressing leakage source localization. Moreover, the dataset uses a single sky background without other interference factors, leaving its generalization capability to complex scenes yet to be validated. Shi et al. [12] combined Faster R-CNN with optical gas imaging technology and employed a model transfer strategy to achieve real-time automatic detection of hydrocarbon leaks. With the rapid advancement of artificial intelligence technologies, specialized models for gas detection [13,14] have been continuously developed. Nevertheless, the performance of deep learning-based detection methods largely depends on large-scale, high-quality, and realistic gas leak datasets for training. Data acquisition requires specialized equipment, and models deployed in field conditions are sensitive to scene variations, often requiring repeated retraining, which limits their practical applicability.

Motion target detection methods based on background modeling do not rely on large annotated datasets for training. Under current conditions where infrared gas datasets are difficult to obtain, such approaches exhibit clear advantages. Moreover, these methods are grounded in well-defined mathematical principles for data processing, providing better interpretability and generalization capability in scene understanding and practical applications. Zuo et al. [15] combined temporal adaptive frame difference filtering, fuzzy C-means clustering, and the Gaussian Mixture Model (GMM) to achieve adaptive segmentation of gas regions. Gao et al. [16] utilized an improved visual background extraction algorithm to locate leakage gas clouds and performed quantitative detection using dual-band differential data. Lu et al. [17] enhanced dark details in infrared images using contrast-limited adaptive histogram equalization (CLAHE) and improved the GMM to achieve adaptive segmentation of gas leaks. Liu et al. [18] enhanced image contrast through gamma transformation and edge suppression techniques, used mean-shift clustering to localize gas regions, and finally detected gas flow direction using grayscale stretching and frame differencing. Although significant progress has been made in applying motion detection methods to infrared gas leak detection, existing approaches still have considerable room for improvement in exploiting the infrared imaging characteristics and diffusion properties of gas.

In previous work [19], the combination of background modeling and density-based clustering was explored to detect gas targets in a single gas leakage scenario. However, the applicability and accuracy of this approach are limited when dealing with complex scenes, particularly in the presence of natural moving object interference. To address the challenges of environmental interference and the blurred, low-contrast characteristics of gas targets in infrared images, this paper proposes a gas leak detection algorithm based on the non-solid characteristics of gas and the spatiotemporal information of infrared image sequences. Specifically, the Gaussian Mixture Model is constrained using the non-solid characteristics of gas to suppress interfering natural moving targets. A multi-scale cross-attention fusion model is introduced to integrate features across different spatial scales and extract fine-grained image details, thereby improving infrared image quality and enhancing detection performance. Finally, a density-based clustering algorithm is employed to cluster gas targets with varying spatial densities in the foreground images, enabling complete segmentation of gas leakage regions.

The main contributions of this paper are summarized as follows:

(1) By analyzing the distinct temporal grayscale variation between gas plumes (smooth transitions) and solid moving objects (abrupt changes), we propose a foreground extraction method constrained by non-solid characteristics, where a dual-criterion constraint is innovatively integrated into the Gaussian Mixture Model (GMM).
(2) A Multi-Scale Cross-Attention Feature Fusion Model with a specialized self-supervised denoising strategy is designed, which forces the network to learn only the sensor and environmental noise distribution while perfectly preserving the weak edge features of low-contrast gas.
(3) A systematic gas leak detection and segmentation framework is constructed for complex scenes. This systematic combination effectively resolves the compounded challenges of solid object interference, noise corruption, and irregular region fragmentation in complex environments, achieving robust and complete segmentation of gas leakage areas.

## 2. Infrared Imaging Gas Leak Detection Experiments

### 2.1. Principle of Gas Leak Detection

**Figure 1:**
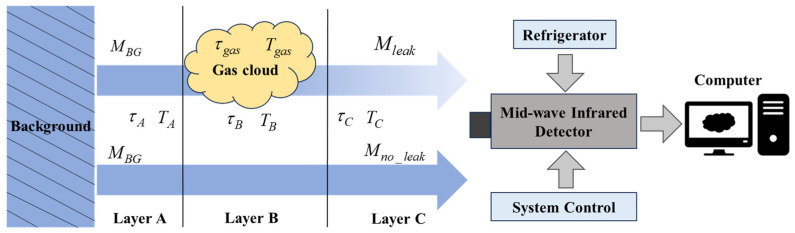
Gas leak infrared imaging detection principle.

The principle of infrared imaging-based gas leak detection is illustrated in Figure 1. The background radiation and reflected infrared spectra are integrated within the response band of the detector to generate an infrared radiation grayscale image of the scene. When gas leakage occurs within the field of view, the presence of the gas plume causes a difference between the radiation absorbed along the gas path and the surrounding environmental radiation. According to the layered radiative transfer model [20], the radiative transfer equations for the gas path and the non-gas path can be expressed as follows:

(1) $$\begin{array}{l} M_{leak}=\tau_{C}\tau_{gas}\tau_{A}M_{BG}+\tau_{C}\tau_{gas}(1-\tau_{A})M_{A}+\tau_{C}(1-\tau_{gas})M_{gas}+(1-\tau_{C})M_{C}\\ M_{no\_leak}=\tau_{C}\tau_{B}\tau_{A}M_{BG}+\tau_{C}\tau_{B}(1-\tau_{A})M_{A}+\tau_{C}(1-\tau_{B})M_{B}+(1-\tau_{C})M_{C} \end{array}$$

where $\tau_{i}$ denotes the atmospheric transmittance of the i-th layer, $\tau_{gas}$ represents the spectral transmittance of the gas, $M_{BG}$ denotes the background radiation, and $M_{i}$ represents the emitted radiation from each layer.

When the radiation difference exceeds the sensitivity threshold of the detector, an infrared image of the leaking gas can be formed. As the gas concentration increases, the absorbed radiation becomes stronger, resulting in more pronounced dynamic variations in the image. In this study, a motion target detection algorithm is employed to process gas leak video sequences, enabling effective detection of leaking gas within the scene.

### 2.2. Experimental Setup and Data Acquisition

The experiments were conducted using a self-developed cooled mid-wave infrared (MWIR) imaging system with a spectral response range of 3.2–3.4 μm. The detector array has a resolution of 320 × 256 pixels with a pixel size of 30 μm, a noise equivalent temperature difference (NETD) of less than 20 mK, and a frame rate of 30 frames/s.

Methane and HFC refrigerant gas were used in the experiments. Methane is a typical representative of industrial gases and exhibits a prominent absorption peak near 3.3 μm [21], which matches the response band of the detector used in this study. However, methane is a flammable gas, and its release during experiments may pose potential safety risks to the experimental environment. Therefore, strict control of methane emissions and necessary safety precautions were implemented during the experimental process. The HFC refrigerant gas is a portable coolant that is colorless, odorless, and highly safe to use. During release, it undergoes rapid vaporization accompanied by significant heat absorption, producing detectable radiation differences within the infrared absorption band. Moreover, the release of HFC gas does not cause significant environmental hazards during experiments. This property helps ensure experimental safety while also satisfying the requirement for large quantities of image data for algorithm validation.

**Figure 2:**
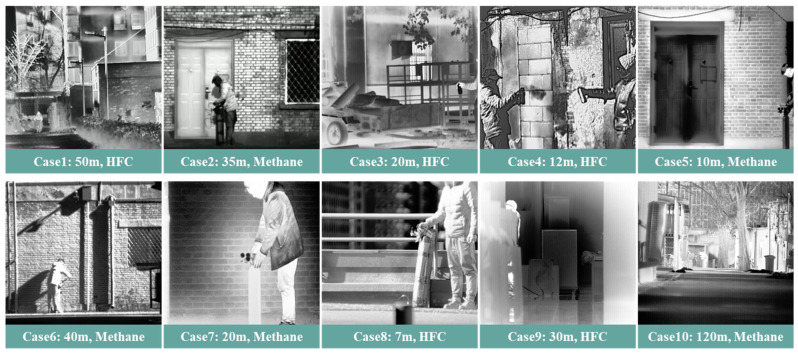
Experimental scenario and representative frames of gas infrared image sequence.

Infrared imaging experiments for gas leakage were conducted under multiple scenarios. The experimental scenes included various interference factors such as pedestrians, vehicles, and swaying trees. Figure 2 presents the experimental scenes used for data acquisition as well as representative frames from the infrared gas image sequences.

This study conducts a comparative analysis of imaging performance under different leakage rates and background conditions. The experimental results indicate that the infrared imaging effect of gas leakage is not directly proportional to the leakage rate. Instead, it is jointly influenced by multiple factors, including the intrinsic properties of the gas, the imaging background, and environmental conditions.

**Figure 3:**
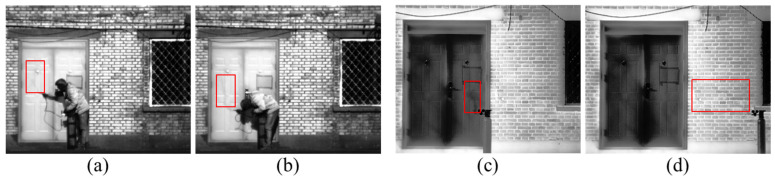
Comparison of infrared imaging for gas leakage. (**a**) 21 January, leakage rate 0.5 L/min; (**b**) 21 January, leakage rate 5 L/min; (**c**) 25 January, iron door background; (**d**) 25 January, brick wall background.

As illustrated in Figure 3, experimental images of the same scene acquired on different dates clearly demonstrate that infrared imaging characteristics vary under different environmental conditions such as ambient temperature, humidity, and illumination intensity. Specifically, Figure 3a,b present infrared images of the same scene with different leakage rates, yet the visual differences in the leakage effects are not significant. In contrast, Figure 3c,d compare leakage images under different background conditions with the same leakage rate. The gas plume is clearly visible against the iron gate background, whereas it is difficult to observe against the brick wall background.

These observations indicate that the grayscale differences observed in infrared images cannot accurately reflect the actual leakage conditions. To a large extent, the infrared imaging performance of gas leakage depends on environmental conditions and the complexity of the background.

Due to the unique characteristics of infrared imaging for gas leakage, the detectability of gas targets in infrared images varies significantly according to visual observation. Based on qualitative assessment, the infrared gas images can be categorized into three levels of detection difficulty:

(a) **Easy:** The gas plume is highly prominent, and the leakage gas can be easily observed in the scene through direct visual inspection.
(b) **Difficult:** The gas plume is relatively subtle and partially concealed, requiring careful inspection of local regions to identify the presence of gas leakage.
(c) **More difficult:** The leaking gas cannot be directly recognized from a single frame. Instead, multiple consecutive frames must be compared to track the position changes of the gas plume in order to identify the leakage phenomenon.

**Figure 4:**
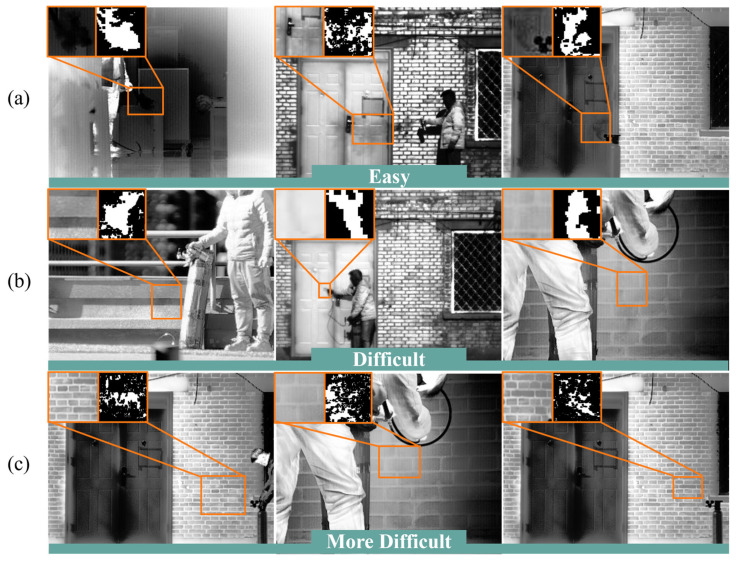
Different visual effects display diagram. (**a**) Easy detection; (**b**) Difficult detection; (**c**) More difficult detection.

Figure 4 presents representative examples of these categories. The enlarged gas regions and their corresponding frame-difference images are shown in the upper-left corner of each image. This study primarily focuses on the difficult detection scenarios, where gas leakage is less visually apparent and therefore more challenging to detect. We captured a total of 5 infrared video sequences in the experiments, including two types of gases: methane and HFC refrigerant. We randomly collected 20 image frames from each sequence as the test data. The leakage rates range from 0.5 L/min to 5 L/min. The scenes include different backgrounds with interferences such as pedestrians, vehicles, and trees.

## 3. Method

**Figure 5:**
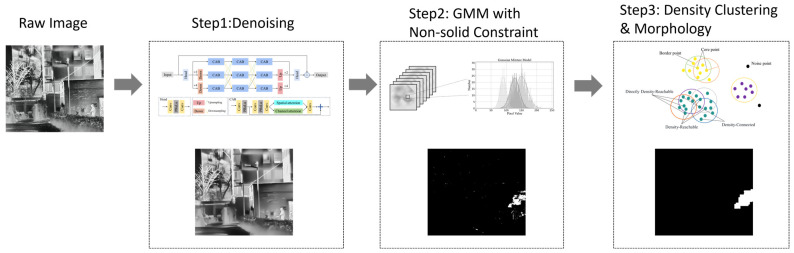
The whole architecture of our method.

This section details the proposed infrared gas leak detection method based on non-solid characteristics and spatiotemporal information, as illustrated in Figure 5. To address the challenges of solid object interference, image noise, and blurred gas edges in complex scenarios, an overall algorithm architecture consisting of three consecutive stages is constructed. First, a Multi-Scale Cross-Attention Feature Fusion Model is employed to denoise the raw input infrared image sequences as a preprocessing step, aiming to suppress sensor and environmental noise, thereby providing high-quality inputs for subsequent foreground extraction. Second, the denoised images are fed into the Gaussian Mixture Model (GMM) for background modeling and foreground extraction. During this process, the non-solid characteristic constraints of gas are incorporated to effectively exclude solid moving interferences such as pedestrians and swaying trees, preliminarily obtaining the gas foreground regions. Third, a density-based clustering algorithm combined with morphological operations is applied to the extracted gas foreground image to filter out isolated noise points and fill regional holes, thus achieving complete segmentation of the irregular gas leakage areas. This overall pipeline ensures the ordered flow and progressive optimization of data from the raw input to the final leakage region segmentation.

### 3.1. Gas Target Detection Method Based on Non-Solid Characteristics

**Figure 6:**
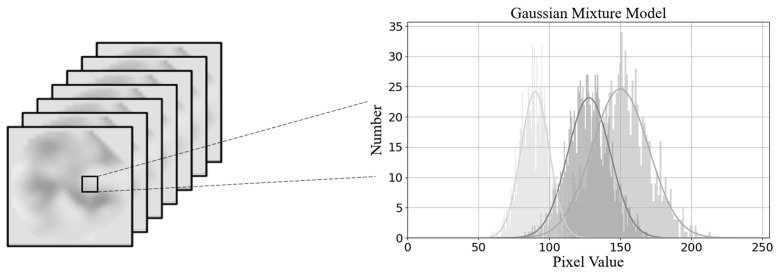
Gaussian mixture model diagram [19].

In motion target detection tasks, the Gaussian Mixture Model (GMM) [22], which exploits temporal correlation information, is a dynamic feedback-based background modeling approach. It can adaptively update the background model according to the matching results in real time, allowing the model to dynamically adjust to complex scenarios such as illumination variations and background disturbances. A schematic illustration of the model [19] is shown in Figure 6.

The GMM represents each pixel in the scene as a linear combination of K Gaussian distributions. Let the probability density function of the Gaussian mixture model for pixel (x,y) at time t be defined as follows:

(2) $$P(f(x,y,t))=\sum_{i}^{K}w_{i}(x,y,t)\cdot N(f(x,y,t),\mu_{i}(x,y,t),\sigma_{i}^{2}(x,y,t))$$

where K denotes the number of Gaussian components; $w_{i}(x,y,t)$, $\mu_{i}(x,y,t)$, and $\sigma_{i}^{2}(x,y,t)$ represent the weight, mean, and variance of the i-th Gaussian component at time t, respectively; and $N\left(f\left(x,y,t\right),\mu_{i}\left(x,y,t\right),\sigma_{i}^{2}(x,y,t)\right)$ denotes the probability density function of the i-th Gaussian component at time t:

(3) $$N(f,\mu ,\sigma )=\frac{1}{\sqrt{2\pi }\sigma }e^{-\frac{{\left(f-\mu \right)}^{2}}{2\sigma^{2}}}$$

(1) **Determination of pixel–model matching.** The new pixel value $f\left(x,y,t\right)$ at time t is matched with all Gaussian components ordered by priority $w_{i,t-1}/\sigma_{i,t-1}$ according to the following criterion. If the matching condition is satisfied, the pixel is classified as background; otherwise, it is classified as foreground.
  (4) $$\left|f(x,y,t)-\mu_{i}(x,y,t-1)\right|\leq 2.5\sigma_{i}(x,y,t-1)$$
(2) **Updating the model weights.** According to the matching result, the weights of the Gaussian components are updated using the following equation. Here, α denotes the learning rate, and $M_{i}(x,y,t)$ is the matching indicator variable that indicates whether the i-th Gaussian component at time t matches the new pixel value $f\left(x,y,t\right)$. If a match occurs, $M_{i}(x,y,t)=1$; otherwise, $M_{i}(x,y,t)=0$. The weights are subsequently normalized according to the updated model weights.
  (5) $$w_{i}(x,y,t)=(1-\alpha )w_{i}(x,y,t-1)+\alpha M_{i}(x,y,t)$$
(3) **Updating the parameters of unmatched models.** For unmatched Gaussian components, the mean μ and standard deviation σ remain unchanged. For the matched component, the parameters are updated using the following equations, where ρ denotes the update rate:
  (6) $$\rho =\alpha N(f(x,y,t)|\mu_{k},\sigma_{k})$$
  (7) μ(x,y,t)=(1−ρ)μ(x,y,t−1)+ρf(x,y,t)
  (8) $$\sigma^{2}(x,y,t)=(1-\rho )\sigma^{2}(x,y,t-1)+\rho {\left(f(x,y,t)-\mu (x,y,t)\right)}^{T}(f(x,y,t)-\mu (x,y,t))$$
(4) **Model ranking and background model construction.** The K Gaussian components are ranked according to the ratio w/σ. The first B Gaussian components that satisfy the following condition are selected to construct the background model:
  (9) $$B=\arg(\min(\sum_{k=1}^{b}w_{k}(x,y,t)>T))$$

In infrared imaging-based gas leak detection scenarios, solid objects such as pedestrians and swaying trees often appear simultaneously with non-solid targets, i.e., leaking gas plumes. Solid objects are characterized by well-defined spatial boundaries and distinct appearance differences from the surrounding environment. In contrast, non-solid targets exhibit shapes that vary over time, lack fixed and clear boundaries, and are easily confused with the background.

**Figure 7:**
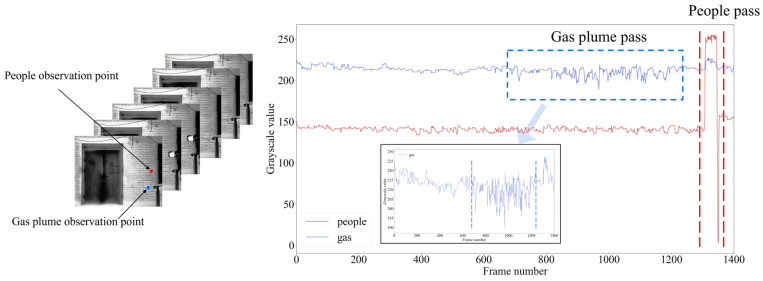
Monitoring point grayscale change curve.

As illustrated in Figure 7, image sequence analysis is conducted by placing monitoring points along the motion trajectories of both a pedestrian target and a gas plume target. The temporal variations of the pixel grayscale values at these monitoring points are recorded. It can be observed that when a pedestrian passes through the monitoring point, the grayscale value undergoes a significant abrupt change. However, when gas leakage occurs, due to the non-solid characteristics of the gas plume, the grayscale variation exhibits a smooth transitional trend, and the magnitude of the change is significantly smaller than the abrupt variation caused by solid objects.

Building upon this observation, this study draws inspiration from shadow detection methods [23,24], where shadow projection mainly alters color saturation while causing minimal changes in hue. In this work, the gas leakage phenomenon in infrared imaging is analogized as a special form of “shadow.” Based on this assumption, the following formulation is employed to process the image and enable the detection of non-solid leaking gas targets.

(10) $$IOP_{t}(x,y)=\left\{\begin{array}{cc} 1 & if\;\alpha \leq \frac{I_{t}(x,y)}{B_{t}(x,y)}\leq \beta \wedge \left|I_{t}(x,y)-B_{t}(x,y)\right|\leq \tau \\ 0 & otherwise \end{array}\right.$$

where $I_{t}(x,y)$ and $B_{t}(x,y)$ denote the pixel values of the input image and the background model at location x at time t, respectively. The parameter 0<α,β,τ<1 represents the constraint parameter. A pixel is classified as a gas foreground target only when the pixel relationship simultaneously satisfies both conditions.

After applying the above target detection algorithm, common natural interference targets in the scene can be effectively suppressed. However, the detection accuracy is still affected by noise interference and the blurred boundaries of gas plumes. Therefore, further optimization of the algorithm is required to improve the detection performance.

### 3.2. Multi-Scale Cross-Attention Feature Fusion Model

In infrared imaging-based gas leak detection scenarios, the disordered diffusion and homogenization characteristics of gas propagation make the concentration distribution and boundary information of gas targets in infrared images highly susceptible to noise interference. This issue is particularly pronounced in low-contrast infrared gas images, where detection algorithms become more sensitive to noise, significantly affecting the accuracy of gas target detection. Therefore, it is necessary to preprocess the raw input images before background modeling to suppress noise interference and enhance gas-related features.

**Figure 8:**
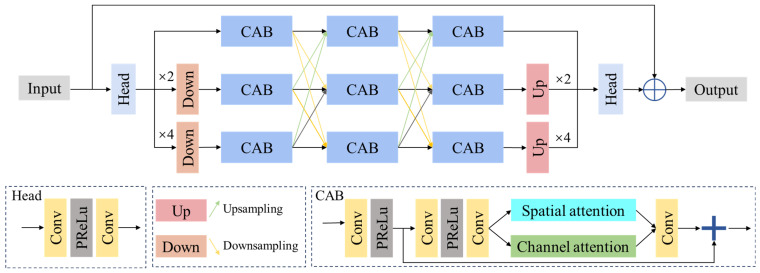
The structure of Multi-Scale Cross-Attention Feature Fusion Model.

To address this issue, this study proposes a multi-scale cross-attention fusion model for improving gas detection in infrared images through image denoising [25], as illustrated in Figure 8. The proposed model adopts a multi-scale cross structure to achieve feature fusion and transformation across different spatial scales. In addition, a Convolutional Attention Block (CAB) is employed to extract local details and contextual features from the images. This design not only improves the quality of infrared images but also enhances the detection performance of the proposed algorithm.

The CAB extract local image features through the short-range dependency convolutional streams, which share information within a feature tensor in terms of both spatial and channel dimensions. The CAB module contains 4 convolutional layers, 2 PreLu functions, and a spatial/channel attention mechanism as shown in Figure 8. The spatial/channel attention module aims to generate a spatial/channel attention map by average and global pooling to rescale the input feature map.

The model adopts an upsampling–downsampling strategy to perform spatial scale transformations on the extracted features. Downsampling is implemented using convolutional layers with a stride of 2, while upsampling is performed using a bilinear interpolation operator. The parallel CAB modules in different scale branches are regarded as a multi-scale cross-fusion unit.

The input features are represented at different spatial resolutions, denoted as $x_{s1}$, $x_{s2}$, and $x_{s3}$, corresponding to scales $S_{1}=1$, $S_{2}=1/2$, and $S_{3}=1/4$, respectively. These three groups of features are first processed by the CAB in their respective scale branches for feature extraction. Through upsampling and downsampling operations, features across different scales are transformed and fused with each other. Finally, linear combinations are applied to generate output features at different scales, which are then propagated to the next cross-fusion unit.

(11) $$\begin{array}{l} x_{CAB_{1}}=F_{CAB_{1}}\left(x_{s_{1}}\right),x_{CAB_{2}}=F_{CAB_{2}}\left(x_{s_{2}}\right),x_{CAB_{3}}=F_{CAB_{3}}\left(x_{s_{3}}\right)\\ y_{s_{1}}=x_{CAB_{1}}+F_{u\times 2}\left(x_{CAB_{2}}\right)+F_{u\times 4}\left(x_{CAB_{3}}\right)\\ y_{s_{2}}=F_{d\times 2}\left(x_{CAB_{1}}\right)+x_{CAB_{2}}+F_{u\times 2}\left(x_{CAB_{3}}\right)\\ y_{s_{3}}=F_{d\times 4}\left(x_{CAB_{1}}\right)+F_{d\times 2}\left(x_{CAB_{2}}\right)+x_{CAB_{3}} \end{array}$$

where $F_{CABi}(i=1,2,3)$ represents the function corresponding to the CAB module at different scales, $x_{CABi}(i=1,2,3)$ denotes the output of each scale branch, and $F_{u\times i}(i=2,4)$ and $F_{d\times i}(i=2,4)$ represent the upsampling and downsampling operations, respectively. $y_{si}(i=1,2,3)$ denotes the output of a multi-scale cross-fusion unit.

For the training of the Multi-Scale Cross-Attention Feature Fusion Model, we captured 10,000 image frames from 10 static non-leakage scenarios using the self-developed cooled MWIR imaging system. The model is trained using a Noise-to-Noise self-supervised learning strategy. Static image sequences from 10 experimental scenarios are selected to construct the dataset, with each sequence consisting of 1000 noisy image frames. Five image sequences are randomly selected as the training set, and the corresponding 5000 noisy images are evenly divided into two groups to serve as the input images and label targets, respectively, thereby satisfying the requirements of self-supervised training. It is worth special explanation that to prevent the model from learning dynamic gas features and consequently over-smoothing low-contrast gas edges, all Noise-to-Noise training data pairs are selected from static background sequences without gas leakage. This strategy enables the CAB module to only learn the random noise distribution of sensors and the environment. During the inference phase, it can realize background purification of the original images, provide clearer input for subsequent GMM foreground extraction, and meanwhile preserve the weak edge features of gas regions.

The experiments are conducted on a Linux system with Python 3.8.10, PyTorch 1.8.1, and an NVIDIA GeForce RTX 3090 GPU. The Adam optimizer is used to update the model parameters during training. The batch size is set to 8, and the initial learning rate is $1\times 10^{-3}$. The model is trained for 200 epochs and optimized using the L1 loss function.

### 3.3. Gas Segmentation Algorithm Based on Density Clustering of Gas Regions

After denoising optimization and gas target detection, the preliminary gas regions in the images are obtained. However, small low-density noise points, which are difficult to remove effectively, may still exist. In addition, due to the non-uniform concentration of gas and environmental interferences, the gas regions in the images exhibit varying densities. Therefore, further processing is required to achieve complete segmentation of the gas regions.

**Figure 9:**
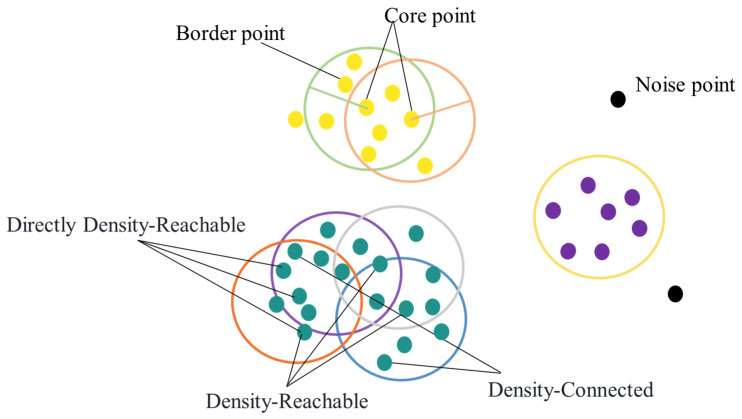
Density clustering diagram.

Since gas regions usually present irregular shapes, traditional clustering algorithms that assume convex clusters (such as K-means) cannot accurately capture their real contours. Therefore, this paper introduces the density-based clustering algorithm DBSCAN [26]. This algorithm determines cluster structures according to the local density of samples, and can effectively identify clusters with arbitrary shapes while removing noise points. The underlying principle is illustrated in Figure 9.

DBSCAN defines core points via the neighborhood radius ϵ and the minimum number of neighborhood points MinPts, and connects density-reachable core points and boundary points into clusters. Taking advantage of the spatial density characteristics of gas foreground pixels, this paper sets appropriate ϵ, MinPts parameters to aggregate discrete gas pixels into complete diffusion regions. Morphological operations are further adopted to fill small holes, thereby achieving complete segmentation of leakage regions.

After applying density-based clustering to the foreground image, clusters are expanded and merged starting from core points, forming maximal regions containing both core and border points. Morphological operations are then applied to fill gaps and connect fragmented regions, resulting in the final delineation of the gas diffusion areas.

## 4. Experimental Results and Analysis

### 4.1. Analysis of the Multi-Scale Cross-Attention Feature Fusion Model

The proposed Multi-Scale Cross-Attention Feature Fusion Model aims to enhance the extraction of infrared gas foreground regions during the background modeling stage. By performing denoising on the raw image sequences, the model effectively suppresses noise while preserving the main image content, reducing confusion between noise and gas leakage in infrared images. As a result, the performance of gas foreground extraction is significantly improved.

**Figure 10:**
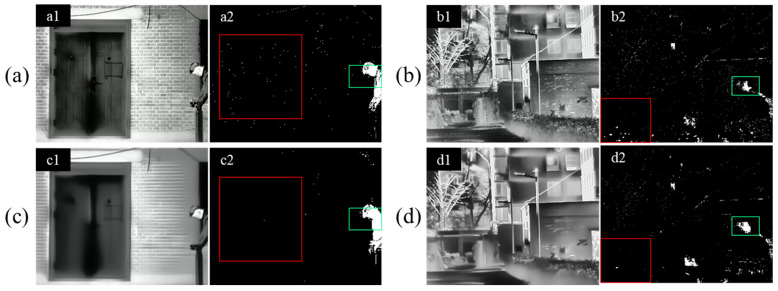
Comparison diagram of ablation experiments for denoising models. (**a**,**b**) show the original image and foreground extraction image before denoising optimization, respectively, while (**c**,**d**) display the original image and foreground extraction image after denoising optimization. Among them, (**a1**–**d1**) denote original images, and (**a2**–**d2**) represent foreground extraction images (rectangular boxes indicate the comparison areas).

Figure 10 intuitively illustrates the original images and corresponding foreground extraction results before (Figure 10a,b) and after (Figure 10c,d) optimization. A comparison shows that, after model optimization, isolated noise points in the extracted foreground are substantially reduced. Observing both pedestrians and gas leakage regions, it is evident that the extracted foreground for pedestrian targets is more complete (Figure 10(a2) vs. Figure 10(c2)), and portions of gas previously obscured by noise are now effectively detected (Figure 10(b2) vs. Figure 10(d2)). These results demonstrate that the optimized model improves the accuracy and completeness of foreground motion target extraction in infrared images.

To quantitatively analyze the effectiveness of the optimized model in this paper for object detection algorithms, the spatial statistical indicator of average nearest neighbor distance is adopted to analyze the pixel spatial distribution in foreground images. Let P be a point set containing n points, d(i,j) be the distance between point i and point j, and $d_{min}(i)$ be the distance from point i to its nearest neighbor. Then the average nearest neighbor distance ${\bar{d}}_{min}$ is calculated as follows:

(12) $${\bar{d}}_{\min}=\frac{1}{n}\sum_{i=1}^{n}d_{\min}(i)$$

where $d_{min}\left(i\right)=\underset{j\neq 1}{\min}d(i,j)$. For each point i, $d_{min}\left(i\right)$ denotes the minimum distance from point i to all other points j(j≠1). The average nearest neighbor distance is obtained by summing the nearest neighbor distances of all points and dividing by the total number of points. A smaller value indicates that the pixel points in space tend to be more closely clustered, implying denser foreground target pixels, thereby reflecting the integrity of the detected regions.

Table 1 presents the validation results of the optimized model performance using the average nearest neighbor distance as the evaluation metric. It can be observed from the comparison that the clustering degree of target pixels in the foreground images extracted by the proposed model is significantly enhanced, which demonstrates the remarkable effectiveness of the proposed model in improving the accuracy of foreground target extraction.

### 4.2. Gas Detection Performance and Clustering Experimental Analysis

**Figure 11:**
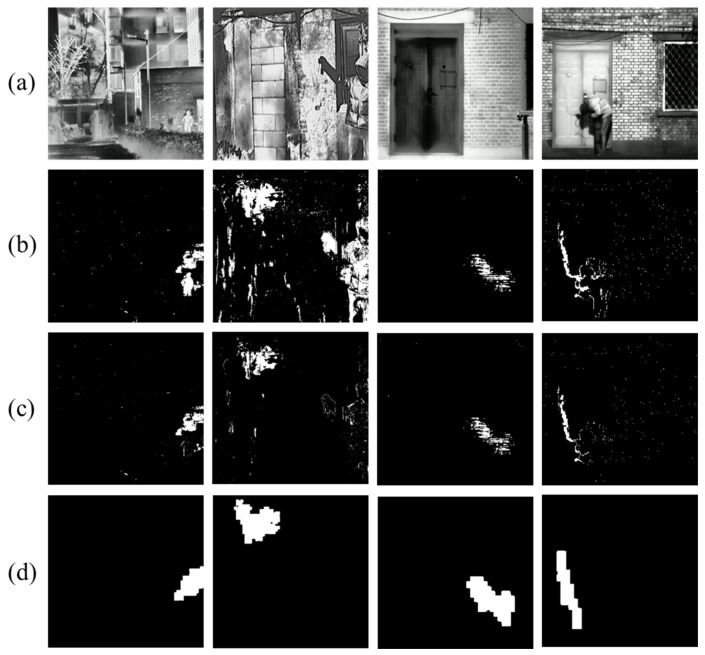
Algorithm processing results. (**a**) Original image; (**b**) Foreground image; (**c**) Gas area; (**d**) Clustering result.

The gas detection algorithm based on non-solid characteristics proposed in this paper eliminates the interference of physical objects during the extraction of foreground moving targets. The density clustering algorithm is adopted to remove noise points from foreground images, ensuring the accurate segmentation of the final gas regions. Figure 11 illustrates the leakage gas detection results under different scenarios. The first row shows the original input frame images; the second row presents the foreground images extracted by the traditional GMM method, in which pedestrians and leaked gas can be observed; the third row displays the leaked gas regions obtained using non-solid characteristics constraints. It can be seen that the proposed constraint method effectively removes the interference caused by factors such as pedestrians and abrupt brightness changes, and roughly obtains the contour of the leaked gas regions. The fourth row shows the final segmented leaked gas regions through density clustering and morphological processing. Combined with the proposed detection optimization model, the experimental results verify that the non-solid characteristics constraint algorithm in this paper can eliminate the interference of physical objects in the scene. Meanwhile, the detection optimization model and density clustering algorithm significantly reduce noise and achieve complete segmentation of the leaked gas regions. In addition, the first and second columns show the detection results of HFC refrigerant gas, while the third and fourth columns present the detection results of methane gas. Experiments demonstrate that the algorithm proposed in this paper has good universality for gas detection.

### 4.3. Comparative Experimental Analysis

**Figure 12:**
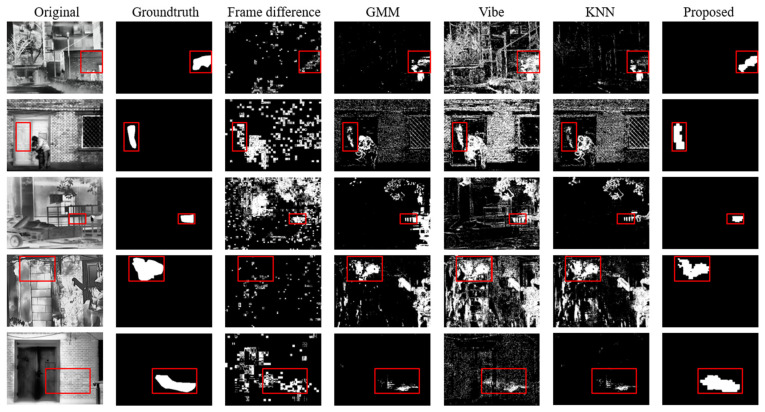
Results of different gas leak detection algorithms.

To verify the effectiveness and advantages of the proposed algorithm for infrared imaging gas leakage detection, experiments were conducted to compare several representative moving object detection algorithms, including the frame difference method, GMM, ViBe [27], and KNN [28]. The experimental comparison is shown in Figure 12. The first column shows the gas images to be detected, the second column presents the ground-truth segmentation images, columns 3–6 display the segmentation results of different comparative algorithms respectively, and the seventh column shows the segmentation result of the proposed algorithm. Given the blurred boundaries and semi-transparent characteristics of infrared gas images, pixel-level manual annotation of ground truth inevitably involves subjectivity. To guarantee evaluation reliability, the Ground Truth (GT) labels in this paper are established following unified criteria: (1) The dynamic diffusion trajectory of gas is observed based on consecutive multi-frame temporal images, rather than relying solely on a single frame; (2) annotation is independently completed by three professionals with rich experience in infrared gas detection. Only pixel regions unanimously agreed upon by the three professionals are retained as the final GT. For pixels where the three annotators disagreed (mainly distributed in the blurry gas edge regions), they were excluded through discussion to ensure high confidence and objectivity of the annotations. Although this strict ‘unanimous agreement’ strategy may slightly shrink the GT edges, it avoids subjective assumptions and ensures the reliability of the comparative experiments.

Combined with the algorithm principles and visual results, it can be concluded that the frame difference method detects changes based on the differences between consecutive frames, making it highly susceptible to noise. Moreover, it frequently suffers from missing holes and missed detection when detecting slowly changing gas leakage processes. The ViBe algorithm performs best in detecting gas regions and can extract gas leakage areas to the greatest extent. However, the problem of noise accumulation in its background update strategy leads to numerous false positives. The GMM and KNN algorithms can better adapt to scene variations by continuously updating the background model, thus achieving satisfactory detection performance. In the horizontal comparison, the main advantages of the proposed algorithm lie in its ability to exclude physical objects in the scene and resist noise interference, enabling accurate segmentation of leakage regions even in low-contrast gas leakage scenarios.

To comprehensively evaluate the algorithm performance, this paper adopts four objective metrics—Precision, Recall, F1-score (F-Measure), and Intersection over Union (IoU)—to quantitatively assess the gas leakage detection regions. Precision and Recall measure the ability of the gas detection algorithm to avoid false positives and false negatives, respectively. False positives lead to misjudgments of leakage conditions, whereas false negatives result in missed leakages, which may cause potential safety hazards. The F1-score is the harmonic mean of Precision and Recall, providing a comprehensive evaluation of the detection performance. IoU reflects the accuracy of detection results by calculating the degree of overlap between the detected regions and the ground-truth leakage regions. The calculated metric results are listed in Table 2.

From the perspective of individual metrics, the GMM algorithm achieves higher Precision than other comparative methods and the proposed algorithm. This is because the GMM tends to ignore low-contrast gas edges to maintain a low false alarm rate, yet it results in severe missed detections. In contrast, the proposed algorithm improves the recall rate of faint gas regions through non-solid feature constraints, with a small number of background false alarms introduced as a necessary trade-off. In industrial leakage early warning scenarios, avoiding missed detections is generally of greater safety value than reducing false alarms.

For comprehensive comparison, the algorithm proposed in this paper exhibits significant superiority in three key metrics: Recall, F1-score, and IoU. This fully demonstrates its superior detection and segmentation capability as well as strong anti-interference performance. Particularly, it performs better in avoiding false negatives, enabling more complete detection of gas leakage and accurate segmentation of diffusion regions, thereby effectively reducing potential safety hazards.

## 5. Conclusions

In the field of infrared detection for industrial gas leakage, the accurate detection of leaking gas and segmentation of its diffusion regions are critically important. To address the challenges of blurred gas leakage contours, low imaging contrast, and susceptibility to external interference in complex environments, this paper employs a self-developed cooled mid-wave infrared imaging system to capture gas leakage videos under various leakage conditions. A novel infrared imaging gas leakage detection algorithm based on non-solid characteristics and spatiotemporal information is proposed. The leakage gas regions are extracted by a Gaussian mixture model detection algorithm constrained by non-solid characteristics. The image sequences are optimized using a multi-scale cross-fusion attention model. Finally, the density clustering algorithm is utilized to achieve accurate segmentation of the leakage gas regions. Experimental results demonstrate that the proposed algorithm can effectively detect and segment low-contrast leakage gas, eliminate interference from physical objects, and resolve the issue of regional holes. It significantly outperforms other comparative algorithms in overall performance. Furthermore, the proposed algorithm is applicable to various types of infrared video data without being restricted by specific imaging equipment, thus demonstrating strong universality. It provides a reliable technical reference for the practical application of gas leakage detection. Future work will further optimize the gas leakage detection method to enhance its robustness in complex interference environments and improve processing speed. Research on multi-source data fusion will also be explored to realize quantitative analysis of gas concentration, providing more precise data support for the early warning and assessment of gas leakage incidents.

## Figures and Tables

**Table 1:** Comparison of average nearest neighbor distance of image pixels before and after optimization (optimal values are in bold).

Method	Case 1	Case 2	Case 3	Case 4	Case 5
Original	1.453	1.477	1.181	1.128	3.307
Proposed	**1.188**	**1.304**	**1.110**	**1.099**	**1.327**

**Table 2:** Comparison of quantitative evaluation results of algorithms (optimal values in bold, suboptimal values underlined).

Method	Case 1				Case 2				Case 3			
	Prec	Rec	F1	IoU	Prec	Rec	F1	IoU	Prec	Rec	F1	IoU
FD	55.9	70.5	60.9	44.3	59.2	39.7	43.4	29.9	65.1	47.7	50.3	39.1
GMM	**64.7**	55.8	59.3	42.3	**91.9**	62.3	74.2	59.0	**92.5**	74.0	82.2	69.8
ViBe	47.1	74.9	57.3	40.5	65.7	89.1	75.6	60.8	80.3	83.5	81.2	69.1
KNN	59.7	56.3	57.4	40.5	83.6	57.1	67.8	51.3	90.7	71.4	79.8	66.5
Proposed	63.7	**90.5**	**74.4**	**59.5**	81.9	**91.5**	**86.3**	**76.0**	82.0	**88.0**	**84.5**	**73.3**
Method	Case 4				Case 5							
	Prec	Rec	F1	IoU	Prec	Rec	F1	IoU				
FD	47.2	56.2	44.2	30.6	42.9	71.2	52.2	35.7				
GMM	**75.8**	76.5	**75.6**	60.9	**82.8**	54.8	65.5	48.8				
ViBe	56.2	**86.8**	67.5	51.5	36.5	70.5	47.3	31.3				
KNN	63.4	84.5	71.7	56.3	78.6	55.4	64.4	47.7				
Proposed	73.9	75.0	74.3	**66.0**	56.1	**93.2**	**68.9**	**53.3**				

## Data Availability

Dataset available upon request from the authors.
